# Natural Fatty Acid Guards against Brain Endothelial Cell Death and Microvascular Pathology following Ischemic Insult in the Presence of Acute Hyperglycemia

**DOI:** 10.3390/biomedicines11123342

**Published:** 2023-12-18

**Authors:** Zaib Ali Shaheryar, Mahtab Ahmad Khan, Huma Hameed, Muhammad Naveed Mushtaq, Sajjad Muhammad, Gamal A. Shazly, Ali Irfan, Yousef A. Bin Jardan

**Affiliations:** 1 Faculty of Pharmacy, The University of Lahore, Lahore 54000, Pakistan; 2 Faculty of Pharmaceutical Sciences, University of Central Punjab (UCP), Lahore 54000, Pakistan; 3 Department of Neurosurgery, University of Helsinki and Helsinki University Hospital, FI-00029 Helsinki, Finland; 4 Department of Neurosurgery, Medical Faculty, Heinrich Heine University, Moorenstrasse-5, 40225 Düsseldorf, Germany; 5 Department of Pharmaceutics, College of Pharmacy, King Saud University, Riyadh 11451, Saudi Arabia; 6 Department of Chemistry, Government College University Faisalabad, Faisalabad 38000, Pakistan; raialiirfan@gmail.com

**Keywords:** neuroinflammation, acute hyperglycemia, ischemic stroke, oxidative stress, brain microvasculature

## Abstract

Ischemic stroke is worsened by the presence of sudden high blood sugar levels, even in individuals without pre-existing diabetes. This elevated glucose concentration hampers the ability of energy-starved brain cells to efficiently use it as a source of energy. Consequently, this leads to the production of abundant amounts of toxic glucose metabolites, which trigger oxidative stress in the brain milieu, particularly in the microvasculature of the brain. A prominent feature of this oxidative stress is the demise of endothelial cells, causing detrimental changes in blood vessels, including a reduction in their vascular diameter, a decreased efficiency of vessel proliferation, and the impaired integrity of tight junctions. These vascular pathologies contributed to an increase in the volume of damaged tissues (infarct), an exacerbation of brain swelling (edema), and a decline in cognitive and motor functions. In a mouse model of ischemic stroke with induced acute hyperglycemia, a naturally occurring saturated fatty acid provides protective cover to the microvasculature by preventing damage related to oxidative stress. Our current research revealed that lauric acid (LA) attenuated infarct volume and reduced brain edema by reducing endothelial cell death, enhancing vessels’ diameter, promoting vascular angiogenesis, and stabilizing barrier functions. Animals administered with this natural compound showed a significant reduction in 4-HNE-positive vessels. In conclusion, natural saturated fatty acids help to preserve brain microvascular functions following ischemic insults in the presence of acute hyperglycemia.

## 1. Introduction

Clinical outcomes of ischemic stroke are predictable and worrisome. One of the many conditions that exacerbate stroke-related mortality and functional outcomes in patients is acute hyperglycemia. Clinical studies have established a definitive relationship between mortality and raised glucose concentrations in patients with ischemic stroke at the time of emergency admissions. Hospital data suggest that in such patients, an average glucose concentration is found to be up to 110 mg/dL or more [1,2]. The underlying pathways leading to acute hyperglycemia during the onset of ischemic stroke are still being investigated. What is also intriguing is that this acute hyperglycemia is even indiscriminately observed in patients with no diabetic history [2,3]. Emergency management strategies thus also focus on acute hyperglycemia during stroke. What is more concerning for clinicians is that administering insulin in these situations leads to hypoglycemia in two-thirds of the total patients, making patients more vulnerable to mortality. Improving stroke outcomes in patients is thus an ongoing fight [4,5]. The European Stroke Organization (ESO), the American Heart Association (AHA), and the National Institute of Neurological Disorders and Stroke (NINDS) emphasize managing acute hyperglycemia during ischemic stroke [6].

The brain microvasculature that forms the tight junctions is the first to be targeted by neuroinflammatory insults following an ischemic stroke. Interrupted oxygen supply and raised glucose levels aggravate the vascular pathology, complicating the therapeutic interventions needed to preserve brain functions [7]. Deranged glucose metabolism leads to the overproduction of reactive toxic glucose metabolites in cells that are part of the brain microvasculature and tight junctions. Prominent among these glucose metabolites are glyoxal (Gx), methylglyoxal (MG), and 3-deoxyglucosone (3-DG). Having a high affinity to react with amino acid side chains on proteins, the reactive metabolites alter protein functions. These altered protein structures, or advanced glycation end-products (AGEs), produce a burst of reactive radicals that target DNA damage and lipid peroxidation. The primary target of this oxidative stress is vascular endothelial cells that make tight junctions in brain regions. Disruptive tight junctions are thus a distinct feature of ischemic stroke pathology. Compromised BBB opens a window for the infiltration of peripheral immune cells, participating in amplified neuroinflammatory damage, fluid leakage (brain edema), and infarct regions [8,9].

Endothelial cell death in the ischemic core and penumbra regions is marked by expressed apoptotic markers, such as cleaved caspase 3. Ischemic stroke triggers the activation of cleaved caspase 3, inducing apoptotic cell death and altering the elasticity, diameter, and density of the microvasculature in the CNS [10,11]. Genetic deletion of cleaved caspase 3 results in the escaped apoptotic death of endothelial cells under oxygen- and glucose-deprived conditions. Similarly, 4-HNE expressions represent cellular oxidative stress. Ischemic stroke results in increased levels of 4-HNE in endothelial cells within the microvasculature of the brain. A rise in the production of oxidative stress following ischemic brain injury is linked to the sudden and sustained deaths of endothelial cells in the microvasculature [12]. Toxic glucose metabolites produced during the ischemic insult produce excess 4-HNE in endothelial cells, causing cytotoxic outcomes in cerebral vascular cells [13].

Lauric acid (LA) is reported in recent studies to have neuroprotective potential in neuroinflammatory degenerative diseases [14,15]. Considering our results from our recent study, where we showed lauric acid (LA) provided neuroprotection in the animal model of Parkinson’s disease, LA’s role in circumscribing neuronal damage following ischemic stroke was investigated. This is because most of the cellular and subcellular neuroinflammatory pathways that aggravate neuronal damage share similarities and origins. In this background, we evaluated the neuroprotective potential of lauric acid (LA) in ischemic stroke. In our study, we evaluated the anti-oxidative effects of lauric acid (LA) on the structural and functional profile of the brain’s microvascular system in a mouse model of acute hyperglycemic ischemic stroke. We also analyzed the effects of lauric acid (LA) on endothelial cells’ viability under oxygen deprivation and excess glucose.

Our data suggest that lauric acid (LA) administration provides neuroprotective cover to microvascular endothelial cells by limiting oxidative stress-induced damage to the tight junctions. We noted improved vessel diameter, density, and angiogenesis in the peri-infarct region of brain lauric acid (LA)-treated animals. These effects are significantly linked to ameliorated infarct area and brain edema. The natural saturated fatty acid was also found to improve the in vitro survival of endothelial cells under oxidative stress. lauric acid (LA)-treated animals further demonstrated improved cognitive and functional outcomes in behavioral tests. Our data concludes that lauric acid (LA) preserves endothelial cell death against oxidative stress, thus preserving barrier integrity during hyperglycemic ischemic stroke.

## 2. Materials and Methods

### 2.1. Animals

In our experimental cohorts, we used male C57BL/6N mice with ages ranging from 10 to 12 weeks. The animals were bred and housed in an institutional animal house at the University of Lahore, Lahore, Pakistan. At the animals’ facility, these mice were kept in IVC Green 500 cm^3^ ventilated cages. Each cage housed a group of four mice. A 12-h light/dark cycle was maintained with a temperature of 23 °C ± 2 °C. Access to food and water remained ad libitum. All animal interventions and procedures were carried out with prior approval from the institutional research ethics committee (IREC-2019-80). Considering good research ethics, we followed the principles of ARRIVE Guidelines 2.0 in our experiments involving animals. Investigators remained blinded, and for the purpose of reporting the data, we followed the ARRIVE guidelines 2.0 (PMCID: PMC7360023). Study materials are available from the corresponding authors upon request.

### 2.2. Ischemic Stroke Model

Ischemic stroke was induced in mice to best describe in vivo acute hyperglycemic-induced ischemic injury. An animal model of permanent occlusion of the distal middle cerebral artery (pdMCAO) was thus used [16,17,18]. For anesthesia, tribromoethanol (15 μL of 2.5% tribromoethanol/g body weight, i.p.) was injected intraperitoneally. Injection (i.p.) of glucose (50 mg/200 uL in normal saline) was carried out immediately after anesthesia in order to raise systemic glucose levels to mimic acute hyperglycemia. Plasma glucose levels were measured using glucometer and chips (Accu-Chek Instant, Roche, MN, USA) before the injection of glucose and 15 and 45 min after the injection of glucose. The procedure involved incising a 1–2 mm tip of animal’s tail, collecting a drop of blood on the tip of the ship and recording the digital measurements (mg/dL). A surgical procedure included a skin incision stretching from the base of the left ear to the left orbit. This followed the removal of the underlying temporal muscle, exposing the skull clearly. A bur-hole was created in the skull to fully expose ‘Y-shaped’ MCA, and it was coagulated from three points using a microbipolar electrocoagulation set (Erbe, Tübingen, Germany, Model ICC 50) under a microscope (Hund, Wetzlar, Germany). After this, the skin incisions were aseptically stitched, and animals were put in a ‘recovery cage’ under a red heating lamp (Philips: R40 Flood Light; 250-watt), where their recovery was closely monitored. To prevent surgical distress, animals were injected with Carprofen (Rimady^®^ Zoetis, Parsippany, NJ, USA) twice daily (5 mg/kg body weight; bd).

### 2.3. Experimental Groups and Dosing

For infarct volume and brain edema measurement, tribromoethanol (15 µL of 2.5% tribromoethanol/g body weight, i.p.) was injected to anesthetize animals before point-of-termination. Brains were harvested and instantly preserved on dry ice. These were later stored at −20 °C until silver staining and further immunohistochemistry analysis. A total of 20 µm thick coronal cryosections were obtained for performing silver staining as per the described protocol [16]. Silver-stained slices on microscopic slides were scanned with the HP Precision Scan Program (300 dpi), and images in TIFF format were processed with ImageJ software (National Institutes of Health, RRID: SCR_002285). The infarct volume was measured as mentioned in our early study [1] using the formula below:

Right Hemisphere + Silver Deficit − Left Hemisphere = Infarct Volume.

In our experiments, the mortality rate hovered at approximately 7%, underscoring surgical consistency.

**Figure 1:**
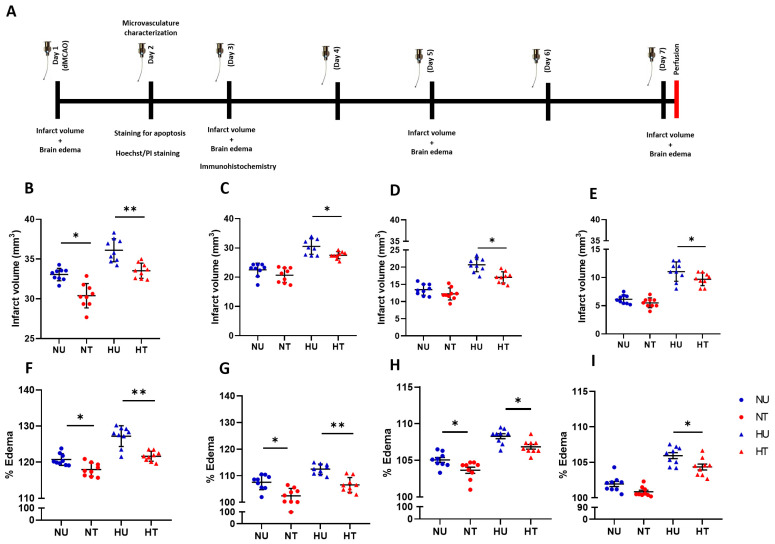
Study design and limiting infarct volume and brain edema in ipsilateral ischemic brain under hyperglycemia in LA-treated animals. (**A**) A description of the study design with time points that represent various stages of ischemic stroke: acute (post-24 h), sub-acute (day 3), moderate (day 5), and chronic (day 7). (**B**–**E**) Silver-stained quantification showing LA limiting the infarct volume in hyperglycemic animals after 24 h, 3, 5, and 7 days following ischemic stroke, compared to the normoglycemic and respective controls. (**F**–**I**) Brain edema attenuation in LA-treated hyperglycemic ischemic-stroke animals at four time points; the values presented are the mean ± SD. The analysis was performed using ordinary one-way ANOVA and Tukey’s multiple comparisons test (**B**–**I**). * *p* < 0.05; ** *p* < 0.01.

A randomization approach to distributing animals into groups was adopted. This put nine animals in each group (*n* = 9). The study included four experimental groups: normoglycemic-treated (NT), hyperglycemic-untreated (HU), normoglycemic-untreated (NU), and hyperglycemic-treated (HT). Treatment groups were orally administered 1.32 mg/kg of LA, while untreated groups were given vehicle doses of Cocos nucifera oil. For determining infarct volume (mm^3^) and brain edema (%), animals were decapitated at four time points after surgery (pdMCAO); days 1, 3, 5, and 7, which represent the acute, sub-acute, moderate, and late phases of ischemic stroke, respectively. LA and vehicle were administered via oral gavage (in treatment and control groups, respectively). The first dose (1.32 mg/kg) was administered 2 h prior to the surgical procedure (day 1), and then this time point was kept constant for daily dosing for different sub-cohorts (3-day, 5-day, and 7-day), as depicted in Figure 1A.

For lauric acid (LA) dosing, we relied on the results of our recent study [14], where we used two doses of lauric acid (LA), i.e., 0.66 mg/kg and 1.32 mg/kg of body weight, via oral gavage. With the later dose (1.32 mg/kg), anti-inflammatory results were more significant. For dosing frequency, we relied on the pharmacokinetic profile of lauric acid (LA) in a study that underscored that, because of its lipophilic nature, lauric acid (LA) shows its peak therapeutic effect in 2 h [19]. These formed the basis for the selection of a specific dose and frequency during the all-experimental procedures.

### 2.4. Immunohistochemistry and Quantification

For the purpose of immunohistochemical staining, 9 animals from each group were sacrificed 72 h post-pdMCAO. Brains were harvested after perfusion with PBS with added heparin (10 IU/mL). Thick coronal brain slices (20 mm) were fixed in methanol (20 °C). For washing, we dipped these sections in PBS and immediately blocked them using 1% BSA in PBS at room temperature. A triple staining was carried out in order to evaluate micro-vessels’ diameter and length (CD31, Col-IV, and DAPI). Similarly, triple staining with 4-HNE, CD31, and DAPI was used for the lipid peroxidation marker 4-HNE. A quick procedure for the CD31 staining included incubating coronal slices with primary antibodies (overnight at 4 °C); occludin: anti-CD31 (557355) 1:1000 (Proteintech, Rosemont, IL, USA); and anti-collagen IV 1:1000 (Abcam, Cambridge, UK). The next day, washing (with PBS) followed incubation with secondary antibodies (conjugated with Alexa 488); rabbit anti-mouse 1:1000, Invitrogen, Waltham, MA, USA; and Goat anti-rabbit (1:1000), Proteintech, respectively. To stain the nuclei, we used DAPI #4083; 1:500; Cell Signaling Technology (Cambridge, UK). For 4-HNE staining, we incubated brain slices with anti-4-hydroxynonenal (4-HNE) 1:1000 (EMD Millipore, St. Louis, MO, USA) and anti-CD31 (557355) 1:1000 (Proteintech, USA). The next day, incubation was carried out with: Anti-4 Hydroxynonenal antibody, 1:100 (Abcam) and Goat anti-rabbit 1:1000, Proteintech, respectively. For immunofluorescence imaging, we used a DMI 6000B Leica microscope (Wetzlar, Germany). Subsequently, fluorescence intensity was measured after processing the images with ImageJ software.

To perform the quantification of vessel length and diameter, we selected an area out of the whole brain-section’s tile-scan. Then, using an auto-thresholding method, we adjusted the Coll-IV threshold and created a mask that was universally applied to all the taken images with CD-31-stained proteins (*n* = 9 per group) in the penumbra region of the Ipsilateral side. We measured the mean intensity values of each image and statistically evaluated them. Likewise, for the analysis of lipid peroxidation markers, 4-HNE-positive vessels and total vessel length were measured in the selected area using ImageJ software (National Institutes of Health), and values were articulated relative to the control mice.

### 2.5. Immunostaining for Apoptotic Marker/Cleaved Caspase-3

Brain endothelial cells make the fundamental structures of the BBB. To study the role of neuroprotective substances in preserving endothelial cell death in vitro, we isolated and cultured primary brain endothelial cells (PBECs) in DMEM medium, as described earlier [20], and divided them into four groups: HU, HT, NU, and NT. For hyperglycemic groups, DMEM was supplemented with a high glucose concentration (4.5 mg/mL) and deprived of oxygen (HGOD) by keeping them in an incubator with 5% CO_2_ at 37 °C. For normoglycemic groups (NU and NT), PBEC cultures were deprived of glucose and oxygen (OGD). The cells were kept incubated with 5% CO_2_ at 37 °C. The treatment groups (HT and NT) were added with a 100 nM concentration of LA for 24 h, while the untreated (HU and NU) groups were added with an equivalent amount of PBS. After incubation for 24 h with LA and PBS, the PBEC groups were challenged with TNF-α (200 mg/mL) for 60 min. Following this, cell cultures were fixed with 4% PFA in PBS and washed with PBS (2 × 5 min). For permeabilization and blocking, 3% BSA in PBS + 0.3% TrX-100 were added and kept at room temperature for 1 h. This followed incubation with primary antibodies: rabbit anti-cleaved caspase-3 (Cat#Asp175; 1:100) and rat anti-CD31 (Cat#550274; 1:200) at 37 °C overnight. The next day, cultures were incubated with secondary antibodies, donkey anti-rabbit IgG Alexa 488 (Cat# A21206; 1:200), donkey anti-rat IgG Alexa 555 (Cat# ab150154; 1:200), and DAPI (1:2000), for 1.5 h at room temperature. Fluorescent microscopic images were then taken, and cleaved caspase3-positive cells were counted using ImageJ software.

### 2.6. Hoechst/Propidium Iodide (PI) Staining

To study the role of neuroprotective substances in preserving endothelial cell death in vitro, we again used the four groups of PBEC cultures mentioned above (HU, HT, NU, and NT). Cells were incubated under the same conditions and treated with LA (100 nM) for 24 h and TNF-α (200 mg/mL) for 60 min again. Cell death in the PBECs was then assessed with Hoechst 33258 dye (Sigma-Aldrich, St. Louis, MO, USA; 10 μg/mL) and propidium iodide (PI; Sigma-Aldrich). Fluorescent microscopic images were taken, and PI-positive cells were counted using ImageJ software.

### 2.7. Lactate Dehydrogenase (LDH) Colorimetric Assay

We also carried out LDH cytotoxic assays in the above-mentioned four groups of PBECs (HU, HT, NU, and NT). This is to evaluate LA’s ability to provide neuroprotection in ECs in vitro by decreasing the release of LDH from the TNF-α-challenged ECs. In the above cohort (Hoechst/PI staining), after treatment with LA (100 nM) for 24 h and TNF-α (200 mg/mL) for 60 min, we collected cell medium (300 uL) from each 12-plate well of all four groups. We measured the LDH release in these samples. Ultrapure water in the cell-culture medium served as a positive control. As per the manufacturer’s protocol (Roche Diagnostics GmbH, Mannheim, Germany), samples were analyzed in duplicate. All these samples (from four groups and the positive control) were incubated with substrate solution (thioridazine HCl), and absorbance at 492 nm was measured for LDH release. Hank’s balanced salt solution (HBSS) was used as a negative control. The relative absorbance values (%) were quantified for analysis.

### 2.8. Western Blotting

To cross-check the immunohistochemistry data, we collected ipsilateral brain (selecting penumbra region) parts (*n* = 6) and carried out immunoblotting as described previously [21]. Protein levels for Caspae-3 were analyzed in brain homogenates. Sodium dodecyl sulfate polyacrylamide gel electrophoresis was used to separate marker proteins in the samples. This followed transferring them to nitrocellulose membranes. A step-wise protocol included; blocking membranes in 5% milk, washing, and a subsequent incubation (4 °C overnight, dark room) with primary antibodies: rabbit monoclonal anti-cleaved caspase-3 antibody (1:1000, Abcam, Cambridge, UK) and rabbit polyclonal antiβ-actin (1:1000, Abcam, Cambridge, UK) (control). The next day, the membranes were washed and incubated with HRP-conjugated secondary antibodies: goat anti-rabbit (1:10,000, Jackson ImmunoResearch Laboratories, West Grove, PA, USA) or goat anti-mouse (1:10,000, Jackson). The detection of markers was carried out using the Fusion Solo S detection system after a chemiluminescence reaction with Pierce TM SuperSignal West Femto Sensitivity Substrate (1:4, Thermo Scientific, Waltham, MA, USA). Here, we normalized cleaved caspase-3 to actin.

### 2.9. Food Intake and Body Weight

Each day, we put pre-weighed food in IVC Green cages, and for food fragments or pallets, a regular inspection was carried out. Using the Sartorius Practum scale, we calculated the food consumed by animals each day for seven consecutive days. Food intake as well as body weight loss (%) were quantified accordingly [22].

### 2.10. Statistical Analysis

To measure the sample size, we conducted power analysis involving the G*Power computation scheme. A *p*-value less than 0.05 was considered statistically ‘significant’ during ANOVA and *t*-test analysis. We did not exclude outliers from the data used for these tests. Here we conducted the Grubbs test (alpha of 0.05) for identifying outliers. Values were expressed as the mean ± SD. Graph Pad Prism version 9.4.1 was used. * *p* < 0.05; ** *p* < 0.01; *** *p* < 0.001; **** *p* < 0.0001. (BS; before surgery; NU; normoglycemia untreated; HU; hyperglycemia untreated; NT; normoglycemia untreated; HT; hyperglycemia treated).

## 3. Results

### 3.1. LA Reduces Infarct Volumes and Brain Edema in Ischemic Stroke Brain, Challenged by Acute Hyperglycemia

LA-administered animals demonstrated decreased infarct volume following pdMCAO, and this trend in decreased infarct volume persisted for all the stages of ischemic stroke (acute, sub-acute, moderate, and chronic). What we found interesting is that the comparable differences in infarct volume between normoglycemic-treated and untreated animals remained non-significant on days 3 and 5 (Figure 1B–E). This could point to the lauric acid’s potential to influence relatively proficiently in limiting expanded oxidative stress that is supplemented under acute hyperglycemia as a result of the production of toxic glucose metabolites. A consistency in the significant difference between treated and untreated mice in the hyperglycemic group even on day 7 suggests lauric acid’s protective effect persists during the late stage of ischemic stroke (Figure 1E). Additionally, the absence of such a difference between treated and untreated normoglycemic animals on day 7 underscores lauric acid’s modulating effects on ischemic damage fueled by hyperglycemia.

Brain edema implies an initial neuroinflammatory response following an ischemic insult to the brain tissues and tight junctions [10]. We thus also assessed the influence of LA on the rate and extent of brain edema formation in acute (day 1), sub-acute (day 3), moderate (day 5), and late stages (day 7) of ischemic stroke. We expected hyperglycemic untreated brains would show expanded fluid leaks compared to LA-treated animals. LA did indeed interfere in edema formation and its expansion in acute, sub-acute, and moderate phases of hyperglycemic ischemic insult (Figure 1F–I). This reduction in edema complemented the infarct volume results (Figure 1B–E). Influence on edema formation is a representative role of LA in modulating oxidative stress under deranged glucose metabolism.

Conclusively, lauric acid (LA) helps in circumscribing the infarct volume in the ischemic brain in a hyperglycemic milieu, particularly at the acute and sub-acute stages of neuroinflammation. How and why remain key questions, and to answer these, we undertook a structured evaluation of microvasculature characteristics.

### 3.2. LA Improved Structural and Functional Characters of Microvasculature in Peri-Infarct Region

**Figure 2:**
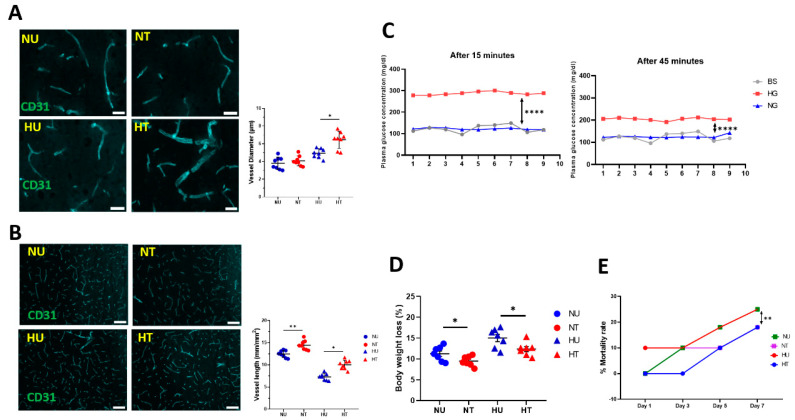
LA improves vascular functions in ischemic stroke under acute hyperglycemia. (**A**) A representative fluorescent microscopic image showing capillaries in the penumbra region of the ipsilateral brain. The tight bar graph shows quantification of CD3-stained vessels for capillary diameter. LA-treated ischemic brain vessels demonstrate a significant increase in diameter compared to untreated brains. Scale bar: 10 µm. (**B**) Representative images (**left**) and quantification of CD31-positive vessels (**right**). LA reduced vessel length in ischemic brains under hyperglycemic and normoglycemic conditions. Scale bar is 100 µm. (**C**) A significant difference in plasma glucose concentration between the normoglycemic and hyperglycemic groups after 15 min (**left**) and 45 min (**right**) of glucose injection. (**D**) LA preserves body weight loss in treated animals. (**E**) LA treatment reduces the mortality rate in animals challenged with ischemic stroke under hyperglycemia. Statistical significance was assessed by ordinary one-way ANOVA and Tukey’s multiple comparisons test (**A**–**E**). Graphs represent means ± SD; * *p* < 0.05; ** *p* < 0.01; **** *p* < 0.0001.

The structural and functional characteristics of microvasculature are broadly affected in the core and penumbra regions of the brain following an ischemic stroke. Vascular pathology directly influences infarct volumes and brain edema following an ischemic stroke. As it is aggravated in the presence of faulty glucose metabolites, we tried to evaluate not only a relationship between vascular pathology and infarct volumes but also the extent to which LA helps revive vascular physiology following pMCAO. For this, capillary diameter and vessel length were measured by measuring CD31-stained vessels and vessel length 48 h after stroke. We found a significant increase in vessel diameter in the ipsilateral hemisphere of the untreated hyperglycemic brains compared to the LA-treated brains. This signifies the collateral circulation in the penumbra that plays a crucial role in reviving blood supply to the infarct core after an ischemic stroke. In the normoglycemic animals (both treated and untreated), this difference remained absent (Figure 2A). Contrary to diameter, vessel length decreases in the ipsilateral ischemic brain compared to the contralateral part. In our study, we compared the length of vessels within the ipsilateral peri-infarct area among normoglycemic and hyperglycemic animals (treated and untreated, respectively). Results suggest that lauric acid (LA) significantly reduced the total vessel length in the treated hyperglycemic brains compared to the untreated animals (Figure 2B), although this effect was seen more in normoglycemic-treated brains. Many factors could influence vessel length, such as the time of occlusion and glucose levels at the time of ischemic stroke. As the pMCAO model permanently occludes the MCA in mice, to exclude the factor of variability in hyperglycemia, we also measured plasma glucose concentrations in hyperglycemic and normoglycemic groups at various time points: before the induction of acute hyperglycemia and 15 and 45 min after the glucose injection. Here, we found a predictably consistent acute hyperglycemia at the time of pMCAO (Figure 2C), suggesting that hyperglycemia remains constant in treated and untreated animals while lauric acid (LA) improves the vessel’s functional properties. This data on acute hyperglycemia remain consistent with our previous data, where, following injection, blood glucose reached its maximum level after 15 min and started reducing after 45 min [23]. In this study, we also found that natural fatty acids preserved body weight loss following pMCAO (Figure 2D) as well as reduced mortality rates in hyperglycemic animals with ischemic insults (Figure 2E). Conclusively, our results suggest that reduced infarct volumes and brain edema in lauric acid (LA)-treated animals are due to improved microvascular characteristics following ischemic brain injury. Next, we evaluated why and how lauric acid (LA) preserved vascular functions under hypoxia and in the presence of deranged glucose metabolism.

### 3.3. LA Reduces Lipid Peroxidation Production in Brain Microvasculature

**Figure 3:**
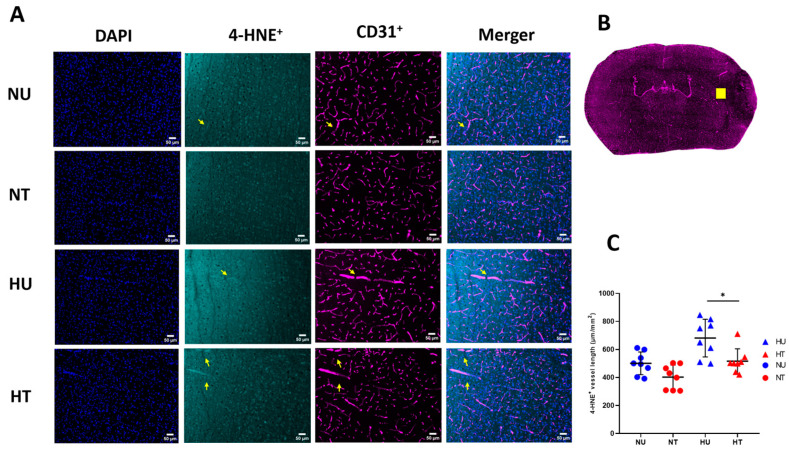
Effects of LA on CD31^+^ 4-HNE^+^ vessels length in ipsilateral brain 72 h-post stroke. (**A**) Fluorescent microscopic images for 4-HNE+ vessel length, colocalized with CD31+; DAPI (blue), 4-HNE (green), CD31+ (magenta), the merger (CD31+ 4-HNE+). (**B**) Quantification of 4-HNE-positive vessel length/total vessel length (**C**) Significant reduction in 4-HNE-positive vessel length (arrows) in the ipsilateral penumbra brain region of treated mice. Analysis followed a two-way ANOVA with Bonferroni’s post hoc test (**C**). Scale bar is 50 µm. Values are expressed as means ± SD; * *p* < 0.05.

Ischemic stroke disrupts the BBB, potentially by affecting the microvascular structures and functions. Disruptive cytoskeletal alterations, increased systemic infiltration, and expressive changes in tight junction-proteins contribute to expanding infarct volume. In the presence of deranged glucose metabolism (inflicted by acute hyperglycemia), these factors are even further compounded because of the oxidative burden. Acute hyperglycemia causes increased oxidative stress, which, in turn, expands cellular lipid peroxidation and its marker 4-HNE, particularly in the endothelial cells of the brain microvasculature, causing cytotoxicity or endothelial cell apoptosis [24]. 4-HNE-induced cellular death affecting barrier functions is well defined [25]. 4-HNE is a stable oxidative lipid peroxidation marker; various pathways clear it from the intracellular milieu to escape cellular damage. We hypothesized that the improvement in vascular characters might be due to either the suppression of the 4-HNE expressions or their increased clearance from the microvasculature endothelial cells by LA. Thus, we analyzed 4-HNE+ vessel length in the ipsilateral side of the hyperglycemic ischemic stroke to see how far, compared to control brains, the 4-HNE+ vessel length varies in the ipsilateral part of the brain. Quantification of 4-HNE-positive vessel length in the immunologically stained ischemic brain sections showed that acute hyperglycemia in stroke increased the number of 4-HNE-positive vessel lengths, while LA administration reduced length. The data showed significantly reduced 4-HNE-positive vessel length in treated hyperglycemic patients compared to controls (Figure 3C). We found more 4-HNE+ vessel length, more oxidative lipid peroxidation in the endothelial cells, and more cellular death and loss of vascular functions. The absence of any significant difference in the 4-HNE-vessle length in normoglycemic-treated and untreated groups suggests acute hyperglycemia worsens the oxidative damage, while LA modulates oxidative pathways powered by toxic glucose metabolites. This suggests that LA plays a neuroprotective role in limiting lipid peroxidative damage to the vascular endothelial cells, either by increased clearance or interfering in their production, thus shielding against BBB disruption. The exact mechanism by which LA interferes in 4-HNE synthesis is elusive; both belong to the fatty acid class and might share cellular substrates or intermediary biomolecular reactants.

### 3.4. LA Administration Shields against Endothelial Cells Apoptosis under Hyperglycemic Ischemic Conditions in PBECs

**Figure 4:**
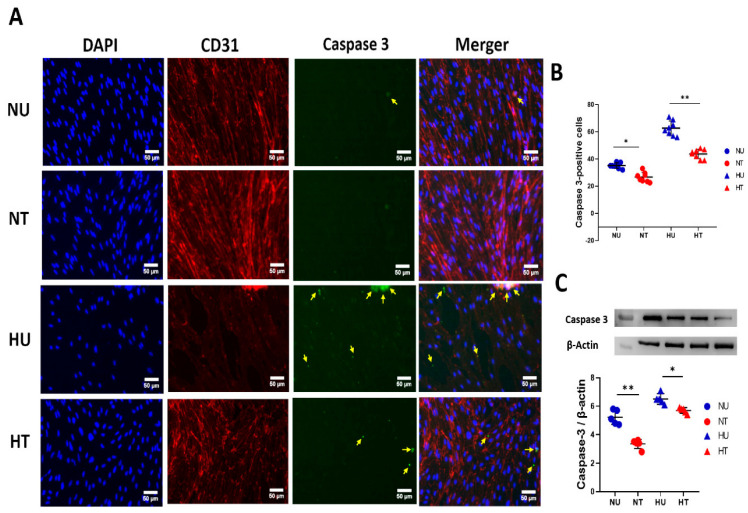
LA shields against apoptosis in PBECs under hypoxic and hyperglycemic conditions, 24 h after LA treatment. (**A**) Representative fluorescent microscopic images of triple staining of PBECs for apoptotic marker, casepase-3 marker; DAPI (blue), CD-31 (red), cleaved caspase-3 (green); the merger. More intense cleaved caspase-3 signals were counted for quantification (pointed yellow arrows) in all four groups of cells. (**B**) Quantification of the number of cleaved caspase-3-positive cells 24 h after LA treatment under hypoxic and glycemic conditions (hyper- and/or normoglycemic). LA-treated NT cells show significantly fewer cleaved caspase-3 fluorescent signals than HU cells. (**C**) Quantification of immunoblotting for cleaved caspase-3 in four groups of PBECs: HU, HT, NU, and NT. LA reduced phosphorylation of apoptotic proteins in the presence of LA in hyperglycemic and normoglycemic groups compared to untreated cells. Analysis followed a two-way ANOVA with Bonferroni’s post hoc test. (**B**,**C**) Scale bar is 50 µm. Values shown as means ± SD; * *p* < 0.05; ** *p* < 0.01.

A brief ischemic stroke triggers complex cellular events that cause death to endothelial cells, thus compromising barrier integrity. Out of many studied pathways, apoptosis is the key mechanism by which endothelial cells die in the brain, and it is exacerbated in the presence of acute hyperglycemia. Thus, we measured expression levels of the apoptotic marker cleaved caspase-3 in PBECs under oxygen-deprived hyperglycemic as well as normoglycemic conditions. LA-treated and untreated cells were statistically compared to see if LA could preserve endothelial cells against apoptosis. A dose of 100 nM concentration of LA significantly reduced the expression levels of cleaved caspase-3 in the HT group compared to HU animals (Figure 4A,B). Though this effect was also present in the normoglycemic group, it remained more pronounced in hyperglycemic-treated brains compared to normoglycemic-treated mice. To confirm our results further, we adopted a more sensitive approach to measuring apoptotic marker expressions: immunoblotting on PBECs (Figure 4C). Here, results complemented staining data; LA reduced casepase-3 levels in HT cells compared to the HU group. Clubbing immunohistochemistry and immunoblotting data; the picture suggests LA influences cellular pathways that cause the apoptosis of ECs in hyperglycemic ischemic conditions (taking one factor here: 4-HNE).

### 3.5. LA Rescues Cells Death in PBECs under Hyperglycemic Ischemic Conditions

**Figure 5:**
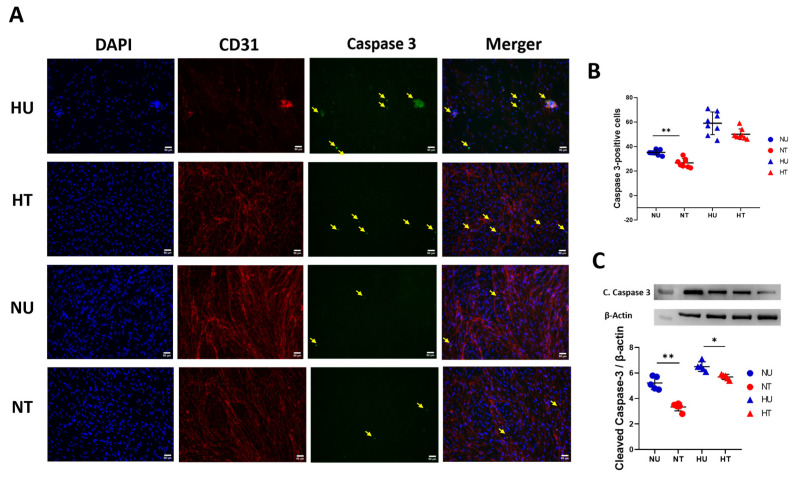
Treatment effect of LA in reducing PBECs death under oxygen deprivation and raised glucose concentration. (**A**) Representative fluorescent microscopic images: Hoechst/PI staining; DAPI (blue), PI (red), and merger. More intense cleaved caspase-3 signals were counted for quantification (pointed yellow arrows) in all four groups of cells. (**B**) Graphic display of quantification data of PI-positive cells in all four groups. Lactate dehydrogenase calorimetric assay quantification, where LA reduced relative absorbance (%) in LA-treated cell medium compared to untreated hyperglycemic and normoglycemic mediums. Statistical significance was assessed by ordinary one-way ANOVA and Tukey’s multiple comparisons test (**B**,**C**). Values are given as means ± SD; * *p* < 0.05; ** *p* < 0.01.

To further examine the hypoxic-induced cell death under reduced oxygen conditions in endothelial cell death, we performed Hoechst/PI staining and LDH cytotoxicity assays. In the immunostaining, we counted PI-positive cells (representation of cell death) in all four groups of cells (HU, HT, NU, and NT) 24 h after LA treatment (Figure 5A). Our results suggest PBECs treated with LA (100 nM concentration) showed fewer PI-positive signals compared to untreated cells (Figure 5B). This significant difference remained more pronounced in normoglycemic cells. To further confirm this annotation, we removed 300 µL of DMEM medium 24 h post-LA treatment. In this medium, we performed an LDH calorimetric assay. LDH release was detected as a measure of cytotoxicity. Absorbance data (percentage relative to the absorbance of control) suggest that LA treatment reduced LDH release in treated cells, both in hyperglycemic and normoglycemic groups, in an oxygen-deprived state compared to untreated cell cultures (Figure 5C). Conclusively, our data underscore that LA rescues cells from death when exposed to oxygen deprivation and excess glucose burden; conditions that mimic acute hyperglycemic ischemic stroke pathology.

## 4. Discussion

In ischemic stroke patients, acute hyperglycemia exacerbates mortality probability and functional and cognitive impairment, primarily due to worsening microvascular pathology. Deranged glucose metabolites increase oxidative stress that causes the death of endothelial cells in brain capillaries, compromising tight junctions and increasing CNS perfusion [26,27]. This association between acute hyperglycemia and endothelial cell death in ischemic stroke has already been established by many studies. Furthermore, BBB disruption expands neuroinflammatory damage to the brain. Thus, targeting barriers’ preservation through effective therapeutic intervention could change the morbidity and mortality statistics to a greater extent. In this study, we evaluated the role of natural fatty acids in shielding against vascular pathology in hyperglycemic stroke. Though LA has already been reported as having neuroprotective effects in neurodegenerative diseases, none of the studies examined its role in hyperglycemic ischemic stroke. In our study, LA demonstrated barrier preservation through shielding microvasculature against oxidative damage. A progressive decline in infarct volumes and brain edema over various phases of ischemic stroke was noted.

Endothelial cells make the functional basis for brain microvasculature, which regulates the delivery of oxygen and essential nutrients and the removal of metabolic waste from the brain. Even a trivial factor can compromise ECs’ functional dynamics, inflicting severe neuronal damage [28,29]. Oxidative stress following an ischemic stroke is one of the many such factors, and acute hyperglycemia extends the scope and intensity of these oxidative radicals. These reactive moieties affect the intracellular molecular structures of ECs, producing disabled metabolites [30]. Levels of the key peroxidation marker 4-HNE are expressed in higher amounts within the ECs of ischemic brain capillaries [31]. Converging evidence suggests preserving cognitive decline in ischemic stroke patients largely depends upon the success of neuroprotective responses that retain microvascular barrier integrity [32]. Our data suggest that LA administration reduced the levels of 4-HNE, signifying improved neurovascular hemodynamics and responses. This effect of LA in protecting against oxidative stress under hyperglycemic conditions is suggestive of its neuroprotective role in ischemic stroke. The exact signaling pathway is doubted; however, studies suggest that, immediately after blood supply occlusion to the brain tissue, lipid peroxidation of unsaturated fatty acids as well as phosphoric acid degradation increases the reactive oxidative burden [33]. These are exacerbated in the presence of increased reactive glucose adducts. A range of in vivo studies suggests that natural neuroprotective medicines downregulate oxidative stress markers, primarily by increasing the levels of anti-oxidant proteins such as heme oxygenase-1 (HO-1), Nrf2, and Akt, as well as by up-regulating GSH and SOD activities [34,35,36]. Microvascular length and diameter are also altered by hyperglycemic ischemic stroke in the peri-infarct region.

Ischemic insult also results in the death of ECs in capillaries, affecting upstream perfusion. The regulation of capillary length and diameter is thus essential for reviving the oxygen and nutrient supply. As a compensation effect, endothelial cells undergo proliferation, and new vessels come into being [37]. In the peri-infarct region, post-stroke acute phase is an important time window, where capillary structures can be shielded against oxidative stress. In our study, LA improved vessel characteristics; increased vessel length and capillary diameter in the peri-infarct region 48 h after hyperglycemic pMCAO. This data complements the studies that suggest that limiting neuronal death acutely after stroke requires passive perfusion by angiogenesis, increased vessel length, and capillary diameter [38]. The exact pathways by which LA preserves vascular characters are still unknown; however, reduced 4-HNE markers, infarct volume, and brain edema point to its potential to circumscribe oxidative stress. Studies prove that JAK2/STAT3, PI3K/AKT, MAPK, and NF-κB signaling pathways are key players in manipulating microvascular tone and subsequent angiogenesis, which is essential for reviving blood and oxygen supply post-ischemic insult. However, suppressing 4-HNE and other reactive aldehydes that target and kill ECs following lipid peroxidation are also cited as potential targets for preserving microvascular functions in stroke [39]. Natural fatty acids interfere with lipid peroxidation. A recent study found tetrahydroxy stilbene glycoside (TSG) activating the GSH GPX4 signaling axis, resulting in a surge in antioxidant activity that downregulated oxidative markers in the brain. In vitro investigations are effective research tools to test hypotheses [40].

In our study, we used primary endothelial cell cultures challenged with oxygen deprivation and hyperglycemia (ODH) to see the reproducible effects of LA in vitro. In these cultures, a monotonous effect can easily be screened out. Like in vivo, ODH causes apoptotic death in PBECs by producing inflammatory oxidative stress. In our study, we measured apoptotic signals in primary endothelial cell cultures to see under which circumstances LA could help escape EC death. Our data suggest that LA reduced cleaved caspase-3 expression in treated cell cultures under both hyperglycemic and normoglycemic conditions. The outcome suggests hypoxia and overproduced toxic glucose metabolites increase the oxidative burden, which in turn causes apoptotic cell death in endothelial cell culture. LA modulates the oxidative burden by shielding against apoptosis. We further consolidated this assertion by quantitatively measuring the number of cells that die in these cultures. Each cell’s death was signified by the PI signal. Results suggest LA decreased the number of cell deaths in these cultures. Furthermore, this in vitro data were complemented by LDH calorimetric assay quantification, where we found relatively increased absorbance in the untreated cell culture medium, compared to the LA-treated culture medium. Conclusively, LA guards against endothelial cell death by limiting oxidative damage that is characteristic of ischemic stroke under deranged glucose metabolism.

## 5. Conclusions

In a mouse model of ischemic stroke with artificially induced acute hyperglycemia, lauric acid (LA) demonstrated its ability to safeguard endothelial cells against oxidative stress in providing neuroprotection. LA effectively reduces the size of the infarcted area and mitigates brain edema, while also enhancing capillary diameter, fostering vascular angiogenesis, and maintaining barrier functions. In the microvasculature of the brain, LA diminishes the production of oxidative markers, thereby lowering the risk of oxidative-induced endothelial cell death. Natural saturated fatty acids play a crucial role in preserving the proper functioning of brain microvessels when confronted with hyperglycemic ischemic insults.

## 6. Scope and Limitation of the Study

Intracellular pathways and signaling mechanisms by which lauric acid (LA) provides such neuroprotection in hyperglycemic stroke needs to be explored. Given the fact that some studies already report receptors on microglia that serve for LA binding, experimental studies involving genetically modified animals and immunohistochemistry analysis can be significant in finding various targets for LA. These new targets for LA can help understand cellular and sub-cellular pathways that lead to neuroprotection in ischemic stroke by this newly reported neuroprotective substance. However, there is limited literature on the mechanisms and signaling pathway involved in acute glycemia resulting in the exacerbation of the ischemic injury in stroke, which presents barriers for selecting suitable and targeted markers with potential neuroprotective LA. With this knowledge, we could not use knock-out animals to verify the exact targets by which LA preserves infarct volume and brain edema in hyperglycemic stroke animals.

## Data Availability

All the data are contained in the manuscript.
